# Study on the Flame Retardancy of Rigid Polyurethane Foam with Phytic Acid-Functionalized Graphene Oxide

**DOI:** 10.3390/molecules28176267

**Published:** 2023-08-27

**Authors:** Xuan Zhou, Feng Jiang, Zhiyu Hu, Faqun Wu, Ming Gao, Zhihua Chai, Yan Wang, Xiaoyu Gu, Yanxia Wang

**Affiliations:** 1 Hebei Key Laboratory of Hazardous Chemicals Safety and Control Technology, School of Chemical Safety, North China Institute of Science and Technology, Sanhe 065201, China; 201701255@ncist.edu.cn (X.Z.); gaoming@ncist.edu.cn (M.G.); chaizhihua@ncist.edu.cn (Z.C.); wangdayanany@126.com (Y.W.); Gxy42@139.com (X.G.); 2 State Key Laboratory of Biobased Fiber Manufacturing Technology, China Textile Academy, Beijing 100025, China; 3 Dean’s Office, North China Institute of Science and Technology, Sanhe 065201, China; wfchljhnyj@163.com; 4 School of Chemistry and Chemical Engineering, Beijing Institute of Technology, Beijing 100081, China; 7520220096@bit.edu.cn

**Keywords:** rigid polyurethane foam, graphene oxide, phytic acid, flame retardancy

## Abstract

A rigid polyurethane foam (RPUF) composite was prepared by compounding phytic acid (PA)-functionalized Graphite oxide (PA-GO) with flame-retardant poly (Ammonium phosphate) (APP) and expandable graphite (EG). The effects of PA-GO on the thermal, flame-retardant, and mechanical properties of RPUF were studied using a thermogravimetric analyzer, a limiting oxygen index (LOI) tester, a UL-94 vertical combustion tester, a cone calorimeter, scanning electron microscopy, and a universal tensile testing machine. The results indicated that there was a significant synergistic flame-retardant effect between PA-GO and the intumescent flame retardants (IFR) in the RPUF matrix. Compared with RPUF-1, the addition of 0.3 wt% PA-GO could increase LOI from 25.7% to 26.5%, increase UL-94 rating from V-2 to V-0, and reduce the peak heat release rate (PHRR) and total heat release rate (THR) by 28.5% and 22.2%, respectively. Moreover, the amount of residual char increased from 22.2 wt% to 24.6 wt%, and the char layer was continuous and dense, with almost no holes. Meanwhile, the loss of mechanical properties was apparently lightened.

## 1. Introduction

Rigid polyurethane foam (RPUFs) is widely used in construction, aviation, refrigeration, and other fields due to its excellent thermal insulation, light weight, high compressive strength, low thermal conductivity, and good corrosion resistance [1,2,3]. However, RPUF is flammable, with a limiting oxygen index (LOI) of only 18%, and can burn quickly after ignition, releasing a large amount of toxic gases [4,5,6], which greatly limits its application. Therefore, the flame-retardant modification of RPUF is necessary. At present, the method of adding flame retardants to RPUF to achieve flame retardancy is relatively common, simple, and easy to implement.

Commonly used flame retardants include intumescent flame retardants (IFRs), halogenated flame retardants, inorganic flame retardants, organic flame retardants, and so on. Among them, IFRs have attracted a great amount of attention due to their advantages of minimal smoke, non-toxicity, and non-dripping properties. In addition, a typical IFR system is composed of an acid source, a carbon source, and a gas source. The classic flame retardant of ammonium polyphosphate (APP), which is widely used in flame-retardant plastics, fibers, and rubber, can usually act as an acid and gas source in the IFR system [7,8,9]

Expandable graphite (EG) is also a typical traditional intumescent flame retardant with excellent flame retardancy. It can serve as a carbon source and act as a condensed phase by forming an expandable char layer at high temperatures [10]. In the flame-retardant studies on the use of EG in RPUF, it was found that EG could make RPUF foam increase and become loose, and the large addition of EG can affect the mechanical properties of RPUF [11,12,13]. In addition, there is a good synergistic effect between EG and phosphate [14,15]. This is mainly due to the fact that phosphate can be decomposed to produce polyphosphate in the heating process, which can participate in the dehydration of EG, thus producing a dense phosphocarbonaceous char layer as a physical protective barrier.

Of course, IFR also has some drawbacks, such as poor dispersion and low flame retardant efficiency. Therefore, researchers often introduce a synergistic flame retardant on the basis of IFR to improve the flame retardant effect [16,17].

Graphite oxide (GO) is a single-atom-thick two-dimensional carbon layer material that has excellent electrical, thermal, mechanical properties and specific surface adsorption capacity and is widely used in microelectronics, energy, catalysis, biomedicine, and other fields. In addition, GO can also be used as a flame-retardant additive for polymers. It has a unique two-dimensional laminar structure that can promote the formation of dense and continuous char layers in the combustion process and isolate the heat transfer and gas exchange with the outside to effectively improve the char residue and flame retardancy of the composite [18,19,20]. However, because of the large van der Waals forces between layers, GO exhibits obvious agglomeration phenomenon and has poor dispersion in the polymer matrix. The oxygen-containing functional groups on the surface of GO can serve as reaction sites to introduce some flame-retardant elements into the skeleton structure of GO, reducing aggregation and improving flame retardancy. For example, GO can be modified with phosphorus [21,22], organosilicon [23,24], IFR [25], and so on. However, few of these chemical modifiers of GO can improve the flame retardancy of RPUF in very small amounts.

As an organic acid, phytic acid (PA) has a high phosphorus content and can improve flame retardancy by promoting the carbonization of polymers [26]. Moreover, PA has been reported to improve the dispersion behavior of its derivatives in the polymer matrix [27].

In this study, PA was used to chemically modify GO to obtain well-dispersed PA-GO. After compounding PA-GO with IFR, the effect of PA-GO on the thermal properties, flame retardancy, and mechanical properties of RPUF composites was studied.

## 2. Results and Discussion

### 2.1. FTIR of GO and PA-GO

**Figure 1:**
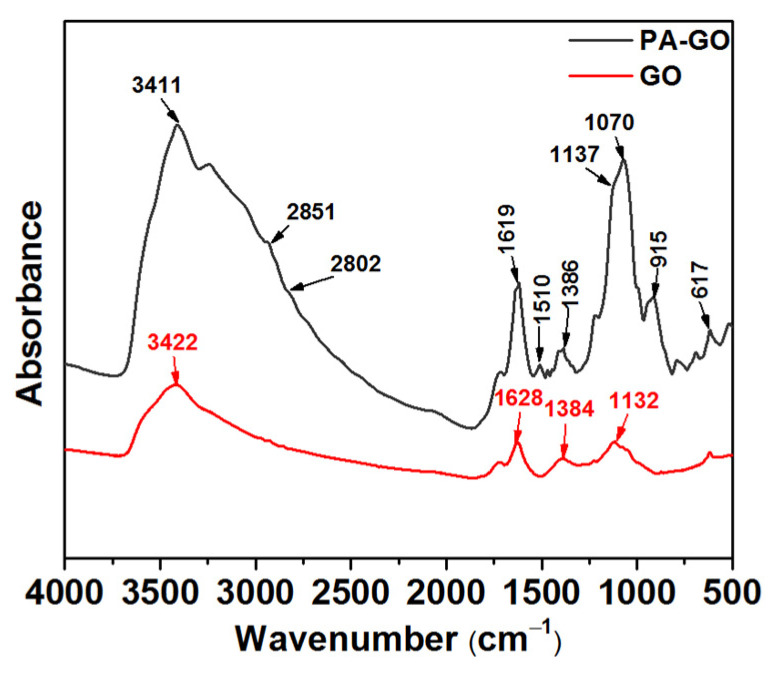
FTIR spectra of GO and PA-GO.

The FTIR absorption spectra of GO and PA-GO are shown in Figure 1. The absorption band at 3422 cm^−1^ corresponded to the stretching vibrations of -OH in GO and PA-GO. The stronger absorption band at 1628 cm^−1^ was the contraction vibration absorption peak of the carbonyl group in -COOH. The bands at 1384 cm^−1^ and 1132 cm^−1^ were associated with the bending vibration of the O-H and C-O-C bonds, respectively. It could be seen that the prepared GO had characteristic functional groups such as hydroxyl, carboxyl, and epoxy groups [28]. As for the infrared spectrum of PA-GO, a new band observed at 2851 cm^−1^ corresponded to the stretching vibration of the methyl group in the silane coupling agent (KH-550), and the peak at 2802 cm^−1^ was the stretching vibration peak of the KH-550 methylene group. At 1137 cm^−1^, it was the stretching vibration of the phospho-oxygen double bond. The peak at 1070 cm^−1^ was the vibration peak of PO_3_^2−^. From the infrared spectrum of PA-GO, it was found that the P=O bonds of phytic acid, PO_3_^2−^ functional groups of phytic acid, and also the characteristic absorption peak of KH-550 were in the infrared spectra of PA-GO, which indicated that phytic acid had reacted with the silane coupling agent and successfully grafted onto the surface of the GO sheets.

### 2.2. SEM of GO and PA-GO

**Figure 2:**
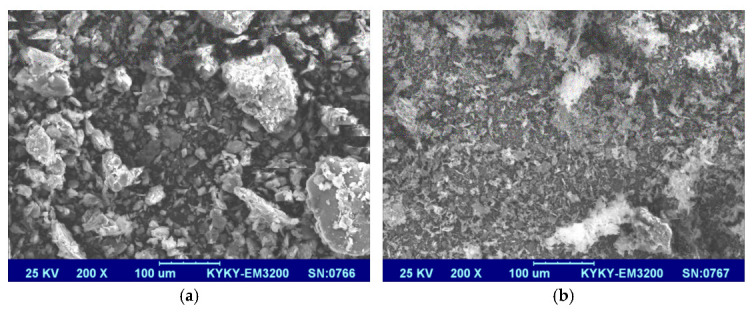
SEM images of (**a**) GO and (**b**) PA-GO.

The microscopic morphology of GO and PA-GO is shown in Figure 2. In Figure 2a, the GO is agglomerated and presents a large number of clumps. Figure 2b shows the morphology of the obtained PA-GO product when modifying GO with PA, and there were few blocky aggregates, and the particles were relatively small. This indicated that PA-GO was more difficult to aggregate compared with GO and that its dispersion has been greatly improved.

### 2.3. TGA of GO and PA-GO

**Figure 3:**
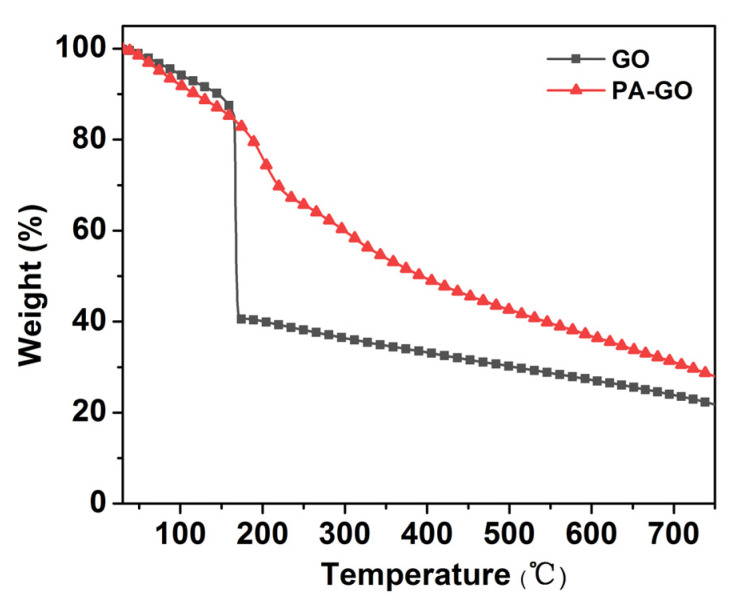
TGA curves of GO and PA-GO.

As shown in Figure 3, the thermal degradation of GO mainly consists of two steps. The first degradation step around 100 °C corresponded to the evaporation of water in the GO sheet. The second degradation step was at 130~750 °C, and the mass loss, which was ascribed to the structural destruction and decomposition of oxygen-containing functional groups such as -OH, -COOH, and C-O-C, and the fracture of -C=C-, was obvious [29]. At 750 °C, the char residues of GO was 23.2 wt%. As for the PA-GO, three degradation steps could be roughly seen. Around 100 °C, only dehydration takes place. The second degradation step was at 130~280 °C, which was attributable the thermal decomposition of some oxygen-containing functional groups. The third degradation step at 280~750 °C was mainly due to the thermal degradation of O-Si-O and the dehydration and carbonization of PA. Finally, there was a 29.8 wt% char residue at 750 °C, which was higher than that of GO.

Overall, it could be seen that the thermal stability of PA-GO was significantly improved compared to GO, and the char residue at 750 °C increased by 22.1% because phosphorus that presented in PA could promote the formation of char through a condensation reaction.

### 2.4. Flame Retardancy

The LOI and UL-94 ratings of RPUF and its composites are summarized in Table 1. From these results, it can be seen that the LOI of the pure RPUF was only 18.4%, and there was no level of UL-94 vertical combustion, which meant that the RPUF was a flammable material and that it burnt out easily in air. After introducing 12 wt% IFR (9 wt% EG and 3.0 wt% APP), the LOI value increased to 25.7%. However, the UL-94 vertical combustion level only increased to V-2 for a long flaming time. Subsequently, the RPUF-2 containing 0.3 wt% GO along with 12 wt% IFR showed a decreased LOI of 25.9% and the same UL-94 V-2 rating. So, the addition of GO did not improve the flame retardant property of RPUF and even slightly reduced the LOI for the poor dispersion in the RPUF matrix. In contrast, the presence of 0.3 wt% PA-GO in RPUF-3 increased the UL-94 rating to V-0 and increased the LOI value dramatically to 26.5%, which was 3.1% and 3.9% higher than that of RPUF-1 and RPUF-2, respectively. This was mainly due to the improved dispersion of PA-GO and the good synergistic flame retardant effect between PA-GO and IFR in the RPUF matrix, resulting in a further increase in LOI and UL-94 rating.

### 2.5. Cone Calorimeter Test

**Figure 4:**
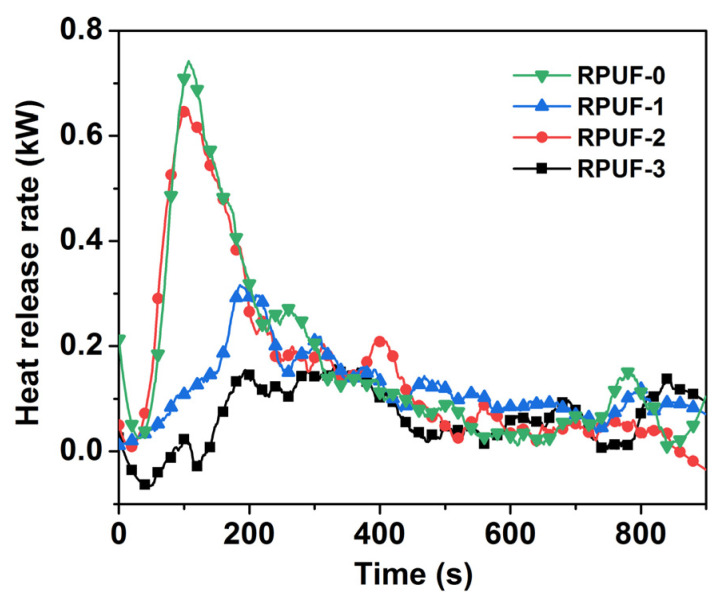
Heat release rate of RPUF and its composites.

**Figure 5:**
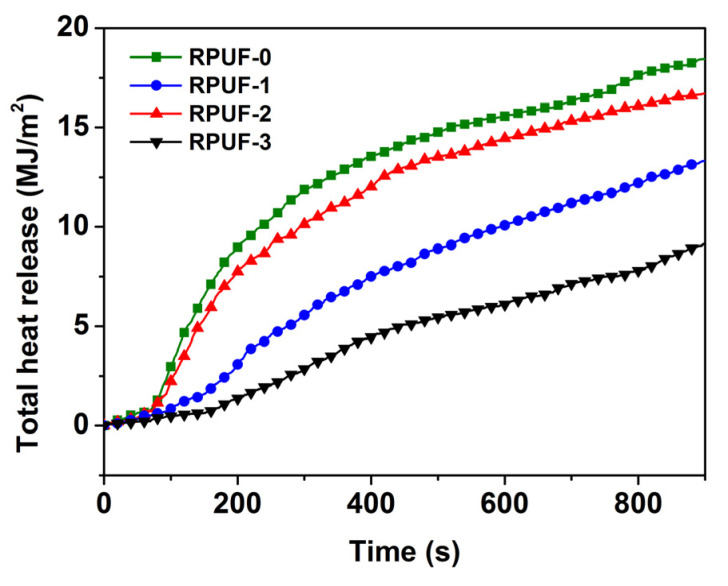
Total heat release of RPUF and its composites.

**Figure 6:**
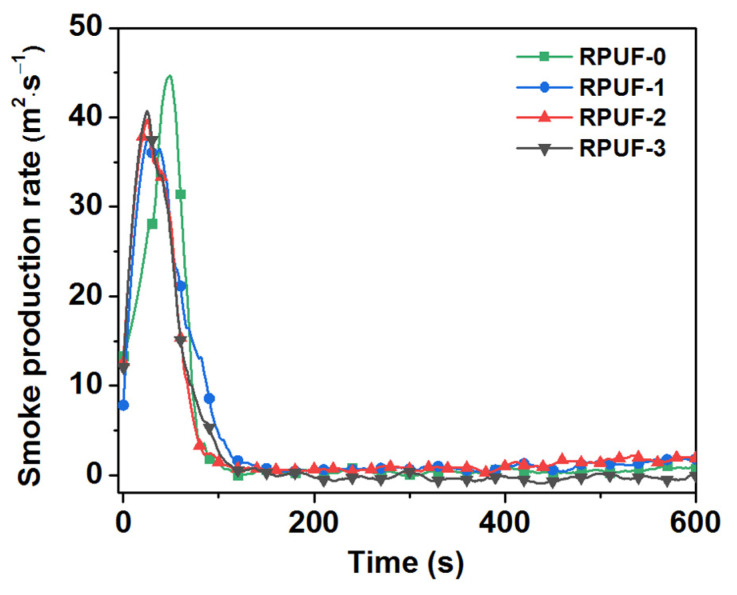
Smoke production rate of RPUF and its composites.

**Figure 7:**
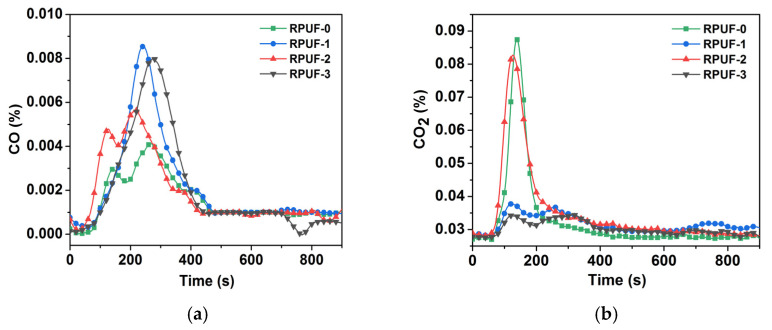
(**a**) CO yield profile of RPUF and its composites; (**b**) CO_2_ yield profile of RPUF and its composites.

As an ideal instrument, the cone calorimeter was used to simulate fire scenarios. Several important parameters, such as the peak heat release rate (PHRR), time to PHRR (T_PHRR_), fire spread index (FSI), total heat release (THR), total smoke production (TSP), peak smoke production rate (PSPR), the peak of carbon monoxide yield (Y_CO_), the peak of carbon dioxide yield (Y_CO2_), and char residue rate are listed in Table 2. The curves of the heat release rate (HRR), THR, smoke production rate (SPR), CO yield, and CO_2_ yield with time are shown in Figure 4, Figure 5, Figure 6 and Figure 7.

As demonstrated in Figure 4, the PHRR of the pure RPUF was 84.32 kW/m^2^ at 107 s, bringing a risk of thermal and fire hazards, while the PHRR of RPUF-1 was reduced to 35.94 kW/m^2^ at 186 s after incorporating 12 wt% IFR. The PHRR of RPUF-2 was reduced to 62.60 kW/m^2^ after incorporating 0.3 wt% GO, and this increased by 42.59% compared with RPUF-1, and the T_PHRR_ of RPUF-2 was decreased to 106 s. So, the addition of a small amount of GO could not reduce the PHRR of RPUF composites but caused a short T_PHRR_, resulting in the rapid burning of the material. On the contrary, the PHRR of RPUF-3 with 0.3 wt% PA-GO was significantly reduced to 18.71 kW/m^2^, and the T_PHRR_ of RPUF-3 was extended to 194 s. Compared with RPUF-0, RPUF-1, and RPUF-2, the PHRR of RPUF-3 clearly decreased by 77.8%, 47.94%, and 70.11%, respectively. Therefore, the addition of modified GO could significantly reduce the PHRR of the RPUF composites and prolong the T_PHRR_ of PRUF. In addition, the fire spread and development of RPUF-3 was relatively slow during the combustion process, and the damage was relatively small, which was due to the synergistic flame retardancy of PA-GO and IFR in the RPUF matrix after the improved dispersion of GO.

FSI was the ratio of PHRR to T_PHRR_, and the smaller the value, the lower the fire risk. As shown in Table 2, the FSI of RPUF-0 was 0.79 kW/(m^2^·s), and that of RPUF-1 decreased to 0.18 kW/(m^2^·s) after incorporating 12 wt% IFR, indicating that IFR could decrease the fire risk to some extent. However, with the addition of 3 wt% GO, the FSI of RPUF-2 increased to 0.6 kW/(m^2^·s) compared to RPUF-1. This illustrated that the addition of GO increased the fire hazard of the RPUF composite. In contrast, with 3 wt% PA-GO, RPUF-3 had a small FSI of 0.10 kW/(m^2^·s). So, RPUF-3 had the highest material safety and the lowest fire risk. The addition of PA-GO significantly improved the fire safety performance of the RPUF composite.

The THR values of RPUF and its composites are shown in Figure 5. At the beginning of the experiment, there was no significant difference in THR between RPUF and its composites. At about 83 s, there began a sudden change in the THR vs. the time curve of RPUF-0 and RPUF-2, with a significant increase in slope and a rapid increase in THR, indicating the rapid combustion of RPUF-0 and RPUF-2. The THR of RPUF-1 only began to rapidly increase around 187 s. The THR of RPUF-3 only began to rapidly increase around 212 s, and the HRR of RPUF-3 was much slower than that of RPUF-0, RPUF-1, and RPUF-2. In addition, the higher the THR, the more heat released by the material during combustion and the poorer the flame retardancy of the material. The THR of RPUF-0 was as high as 18.46 MJ/m^2^, while the THR of RPUF-1 significantly decreased to 13.33 MJ/m^2^. This was mainly because the expanded char layer formed by RPUF-1 could effectively hinder the heat transfer and release. The THR of RPUF-2 with 0.3 wt% GO decreased to 16.71 MJ/m^2^, which was 9.48% lower than that of RPUF-0 and 25.35% higher than that of RPUF-1. So, the addition of GO could increase the THR of RPUF, which was unfavorable to flame retardancy. On the contrary, the THR of RPUF-3 with 0.3 wt% PA-GO was reduced to 9.16 MJ/m^2^, which was 50.39% lower than that of RPUF-0 and 31.27% lower than that of RPUF-1, indicating that the addition of PA-modified GO improved flame retardancy. In addition, RPUF-3 showed better flame retardancy, which indicated that PA-GO had obvious synergistic flame retardancy with IFR in the RPUF matrix after the dispersity of GO was improved.

Smoke is an important cause of death with respect to fires [30]; thus, total smoke production (TSP), smoke production rate (SPR), Y_CO_, and Y_CO2_ are four important parameters in evaluating fire hazards. As listed in Table 2 and Figure 6 and Figure 7, the TSP value of RPUF-0 was 2589 m^2^/m^2^, and the smoke released had a high PSPR value of 64.89 m^2^/s. When IFR was added, the TSP values of RPUF-1 and RPUF-2 increased to 3457 m^2^/m^2^ and 3276 m^2^/m^2^, respectively. However, only the TSP value of RPUF-3 decreased to 2499 m^2^/m^2^. Moreover, the PSPR values of RPUF-1, RPUF-2, and RPUF-3 were reduced to 50.37 m^2^/s, 56.79 m^2^/s, and 50.53 m^2^/s compared with RPUF-0. Specifically, the PSPR value of RPUF-3 decreased by 22.13% compared with RPUF-0. The possible reason for this is that RPUF-3′s more appropriately formed char layer can inhibit the escape of smoke particles generated by the decomposition of the RPUF composites.

The Y_CO_ of RPUF-0 was 0.004%, and that of RPUF-1, RPUF-2, and RPUF-3 increased to 0.009%, 0.006%, and 0.008%, respectively. Among them, The Y_CO_ of RPUF-1 and RPUF-3 increased significantly compared with RPUF-0 for insufficient combustion. In addition, the Y_CO2_ values of RPUF-0, RPUF-1, RPUF-2, and RPUF-3 were 0.089%, 0.039%, 0.084%, and 0.035%, respectively. So, the Y_CO2_ values of RPUF-1 and RPUF-3 were relatively lower also for insufficient combustion, and this result was consistent with the Y_CO_ result. Therefore, PA-GO synergy with IFR could reduce the spread of fire, resulting in the incomplete combustion of RPUF.

Furthermore, the char residue of RPUF-0 was only 4.0%, indicating that the pure RPUF had a poor charring ability. RPUF-3 left 24.6% char residue after combustion, which was increased by 83.7% compared to RPUF-0, increased by 9.8% compared to RPUF-1 (22.2%), and increased by 26.0% compared to RPUF-2 (18.2%). This indicated that PA-GO and IFR had an obvious synergistic effect on facilitating the carbonization of the RPUF matrix and that the formed char layer could suppress the release of smoke and toxic gases effectively.

### 2.6. Thermal Degradation

**Figure 8:**
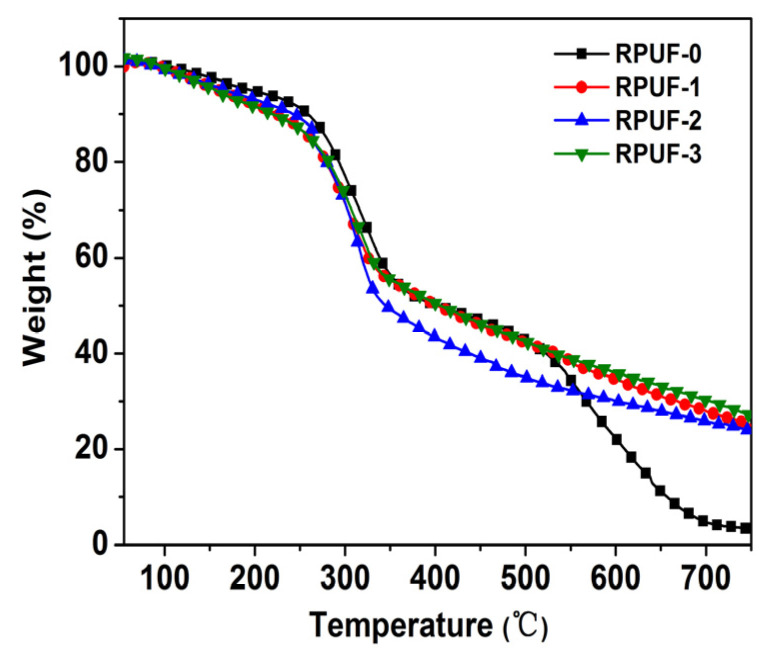
TG curves of RPUF and its composites.

The thermogravimetric analysis curves of RPUF and its composites in nitrogen and shown in Figure 8, and the related data are listed in Table 3. The thermal degradation of RPUF-0 in nitrogen could be divided into three stages. The first stage was within 110~140 °C, which was mainly due to some mass loss caused by the volatilization of water vapor in the sample. In the second stage (240~450 °C), the carbamate bonds in the polyurethane molecule were broken; small molecules such as diisocyanate, alcohol, and carbon dioxide were generated; and some diisocyanate may have reacted to form diimide [2]. The third stage was within 450~750 °C, mainly due to the degradation of substituted urea, which was generated by the reaction of diimide carbonate with alcohol or water vapor [2]. At this stage, the char formation tended to be more stable, and the char residue at 750 °C was about 3.3 wt%.

In addition, as shown in Table 3, the initial decomposition temperature (T_-5 wt%_) of RPUF-0 was 198 °C, while the T_-5 wt%_ of RPUF-1, RPUF-2, and RPUF-3 decreased to 161 °C, 168 °C, and 156 °C, respectively. In other words, the T_-5 wt%_ of the RPUF composites significantly decreased after adding flame retardants. Moreover, with increasing temperature, when RPUF-0 lost 10 wt%, the corresponding temperature was T_-10 wt%_ (259 °C), while the T_-10 wt%_ of RPUF-1, RPUF-2, and RPUF-3 decreased to 224 °C, 242 °C, and 219 °C, respectively. Therefore, all RPUF composites degraded earlier than RPUF-0, especially RPUF-3. This might be attributed to the decompostion of APP in IFR, and phosphoric acid and ammonia were produced in this process. However, the T_-50 wt%_ of RPUF-2 was 345 °C, which was significantly lower than RPUF or the other RPUF composites. Also, as shown in Figure 8, RPUF-2 decomposed significantly earlier than the other materials when the temperature exceeded 300 °C. This shows that the addition of IFR can promote the early decomposition of RPUF, and the addition of GO accelerated this process. In contrast, the T_-50 wt%_ of RPUF-1 was 403 °C, which is the same as the T_-50 wt%_ of RPUF-0 and close to the T_-50 wt%_ of RPUF-3 (406 °C). Therefore, RPUF-1 and RPUF-3 had higher thermal stability than RPUF-0 after a weight loss of 50%. In addition, the decomposition products were dehydrated and cross-linked into the char layer in the presence of phosphoric acid, and the char residues of RPUF-1, RPUF-2, and RPUF-3 reached 25.0 wt%, 23.7 wt%, and 27.0 wt%, respectively, at 750 °C. Among them, the char residue of RPUF-2 was significantly higher than that of RPUF-0, but it was 5.5% lower than that of RPUF-1. While the char layer must be produced earlier to better protect the matrix and play a role in resistance [31], here, the char layer produced by RPUF-2 was obviously not early enough, resulting in even fewer char layers than in RPUF-1. In contrast, the char residue of RPUF-3 was 87.8%, 7.4%, and 12.2% higher than that of RPUF-0, RPUF-1, and RPUF-2, respectively. This indicates that there was a good synergistic effect between PA-GO and IFR in promoting the rapid crosslinking of the matrix into char residue. Compared with GO, PA-GO has high thermal stability and good dispersion in RPUF, making the synergistic effect of promoting carbonization more significant.

### 2.7. Micromorphology of Residual Char

**Figure 9:**
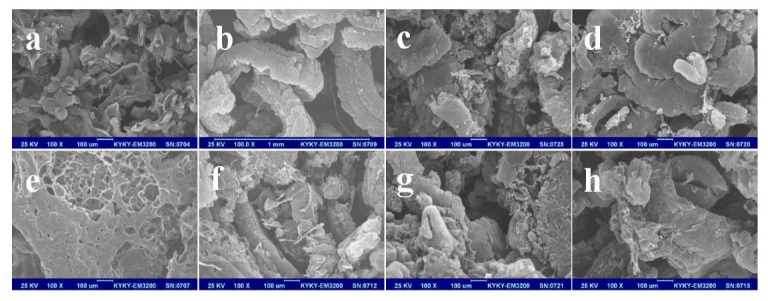
SEM images of residual char after CCTs. Herein, (**a**–**d**) are the outer surface of residual char for RPUF-0, RPUF-1, RPUF-2, and RPUF-3; (**e**–**h**) are the inner surface of residual char for RPUF-0, RPUF-1, RPUF-2, and RPUF-3.

Figure 9 displays SEM images of residual char after CCT tests. The SEM images shown in Figure 9a–h (both at 100×) were used to evaluate the micro morphology of the outer surface and inner surface of residual char for RPUF and its composites, respectively.

As shown in Figure 9a,e, a large number of holes on the inner surface of the char layer of RPUF-0 and the outer surface of the char layer was fragmented, discontinuous, and loose. Such a char layer was too poor to resist the erosion of heat and combustible gas. After the addition of IFR, the internal and external char residue structure of RPUF-1 changed greatly. For the presence of EG in IFR, a large number of vermicular char layers were present, with an obvious vermicular outline and a complete structure, and the char layer was compact. However, after adding GO, the char layer structure in RPUF-2 was incomplete and fragile, with obvious grooves on the inner surface, resulting in a decrease in the quality of the char layer. When PA-GO was added, not only the wormlike char layer was clearly visible in RPUF-3, but both the inner and outer surfaces of the char layer were complete and very dense, and the outer surface of the char layer was very solid with almost no holes and almost no damage, and the quality of the char layer significantly improved. Such a char layer could better protect the RPUF matrix from the erosion of external heat and gas. Therefore, RPUF-3 had the best flame-retardant effect, which was attributed to the better synergistic flame retardancy between PA-GO and IFR in RPUF, and the results were consistent with those of TGA and CCT.

### 2.8. Proposed Flame-Retardant Mechanism

Combined with the above analysis, the flame-retardant mechanism of the RPUF-3 composite was proposed. After combustion, EG acted on the condensed phase, producing a dense char layer on the surface of the RPUF, which limited heat and mass transfer to the polymer and impeded oxygen penetration. At the same time, APP decomposed to produce polyphosphate and ammonia gas during combustion. Polyphosphates could promote the rapid cross-linking and carbonization of the RPUF matrix, while ammonia could promote the expansion of the char layer and dilute the combustible gas. In addition, with the expansion of the char layer, the GO uniformly dispersed in the RPUF matrix could migrate to the surface of the char layer to strengthen the char layer [32], and finally, a dense and continuous expanded char layer was obtained.

### 2.9. Mechanical Properties

The mechanical properties of RPUF and its composites are shown in Table 4. In general, a major disadvantage of conventional phosphorus-based and nitrogen-based organic or inorganic flame retardants is the deterioration of polymer mechanical properties, whether through physical blending or covalent grafting into RPUF [33,34]. Such is the case in RPUF and its composites. After adding IFR in the RPUF system, the mechanical properties of RPUF-1 showed significant decreases in tensile strength, elongation at break, and compressive strength by 50.0, 39.6, and 65.5%, respectively, compared with pure RPUF. When 0.3 wt% GO was added to the flame-retardant RPUF system, the tensile and compressive strength decreased slightly due to the poor compatibility of GO. In contrast, with 0.3 wt% PA-GO incorporation, RPUF-3 increased by 12.5% in tensile strength from 0.08 to 0.09 MPa, 18.8% in elongation at break from 23.26% to 27.63%, and 39.5% in compressive strength from 1.19 MPa to 1.66 MPa. So, the addition of PA-GO improved the mechanical properties of the flame-retardant RPUF composites, which indicated that PA-GO could exhibit excellent nano reinforcement and weaken the damage of flame retardants to the mechanical properties of RPUF composites under the premise of uniform dispersion.

## 3. Materials and Methods

### 3.1. Materials

Polyether polyol (type:1012a) and isocyanate (type:1012b) were purchased from Shenzhen Keshengda Trading Co., Ltd. (Shenzhen, China). Concentrated sulfuric acid (98%, AR), potassium permanganate (AR), and sodium nitrate (AR) were supplied by the Beijing Chemical Factory (Beijing, China). Hydrogen peroxide (30%, AR) and anhydrous ethanol (AR) were bought from the Tianjin Yongda Chemical Reagent Co., Ltd. (Tianjin, China). Phytic acid (PA) and the silane coupling agent KH550 (GR) was bought from the National Pharmaceutical Group Chemical Reagent Co., Ltd. (Shanghai, China). Ammonium polyphosphate (APP) was purchased from the Tangshan Yongfa Flame Retardant Material Factory (Tangshan, China). Graphite powder was bought from the Tianjin Zhiyuan Chemical Reagent Co., Ltd. (Tianjin, China). Expandable graphite (EG, type: ADT150) was purchased from the Shijiazhuang Kepeng Flame-retardant Material Factory (Shijiazhuang, China).

### 3.2. Preparation of GO

The GO was prepared via Hummers’ method [35]. A total of 69 mL 98% concentrated sulfuric acid was added into a 1000 mL beaker under ice bath conditions, and 3.0 g graphite powder and 1.5 g sodium nitrate were added under stirring at 0 °C. After full stirring, 9.0 g potassium permanganate was slowly added in batches, and then the solution temperature was heated to 35 °C for 30 min. Then, 138 mL of deionized water was slowly added, and the temperature was rapidly raised to 98 °C. After reaction for 15 min, the heating was stopped, and the beaker with the solution was cooled to room temperature in a water bath. Then, 15 mL of H_2_O_2_ solution and 420 mL of deionized water were added successively, and the target product was filtered, washed, and dried at 60 °C for 48 h.

### 3.3. Preparation of PA-GO

**Scheme 1:**
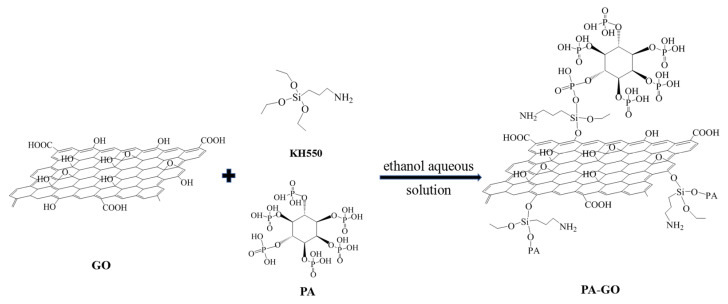
Schematic illustration for the synthetic route to PA-GO.

A total of 2.5 g GO was added to 100 mL 90% ethanol aqueous solution with ultrasonic dispersion at 25 °C for 1 h. Then, 2 mL of the silane coupling agent KH-550 was dissolved in the GO solution via uniformly stirring for 30 min. Subsequently, 0.5 g PA was slowly added and stirred at 500 rpm at 25 °C for 1 h. Finally, it was filtered, washed, and dried to obtain PA-GO. A schematic illustration for the synthetic route to PA-GO is listed in Scheme 1.

### 3.4. Preparation of RPUF Composites

A certain amount of APP, EG, and GO/PA-GO were added to the isocyanate in a beaker via stirring at 300 rpm for 30 min. The polyether polyols were then poured into the previous mixture via stirring at 300 rpm until a uniform mixture was obtained after around 30 s. Finally, the mixture was placed in an open mold to foam for 12 h at room temperature. The raw material compositions of the RPUF composites are described in Table 5.

### 3.5. Characterization

Fourier-transform infrared (FTIR) spectroscopy was conducted using an FTS 2000 FTIR (Varian, Palo Alto, CA, USA) from 4000 to 400 cm^−1^. The oxygen index (LOI) was measured using a JF-3 oxygen index tester (Jiangning, China) in accordance with the ASTM D2863-97 standard. The specimen size for the LOI test was 100.0 × 6.5 × 3.0 mm^3^, and the LOI measurement for each specimen was repeated for three times. The UL-94 rating was obtained by using a PX03001-02 vertical combustion tester (PHINIX, Suzhou, China) in accordance with ASTM D3801-96. The specimen size was 100.0 × 13.0 × 3.0 mm^3^, and the measurement for each specimen was repeated for three times. Cone calorimeter measurements were performed using a cone calorimeter (CONE) (PX-07-007, Phoenix Quality Inspection Instrument Co., Ltd., Suzhou, China) according to the ISO 5660 standard under a heat flux of 35 kW/m^2^. The specimen size was 100.0 × 100.0 × 3.0 mm^3^, and the measurement for each specimen was repeated for three times. Thermogravimetric analysis (TG) was carried out by using a HCT-2 thermal analyzer (Beijing Hengjiu Scientific Instrument Factory, Beijing, China) from 50 to 750 °C with a heating rate of 10 °C˙min^−1^ in a nitrogen atmosphere. The char formed after CONE testing was first sputter-coated with a conductive layer and then observed using a scanning electron microscope (SEM, KYKY-EM3200, Beijing, China) with a 20 kV accelerating voltage. Tensile strength was measured using a CMT4204 electronic universal material testing machine (Meters Industrial Systems Co., Ltd., Shanghai, China) in accordance with ASTM D3574. The compression test results were also measured using an electronic test machine in accordance with ASTM D1621-94 with a compression rate of 2 mm/min at room temperature. The specimen size was 50.0 × 50.0 × 50.0 mm^3^, and the measurement for each specimen was repeated for five times.

## 4. Conclusions

PA-GO was successfully prepared by modifying GO using PA and KH-550 and was introduced into RPUF to increase its flame-retardant properties. The RPUF-3 composites with 12 wt% IFR and 0.3 wt% PA-GO could pass the UL-94 V-0 rating, and its LOI value (28%) was higher than that of the other RPUF composites. Compared with RPUF-1, the PHRR decreased from 33.78 to 19.60 kW/m^2^, the THR decreased from 13.33 to 9.16 MJ/m^2^, and the TSP also dropped from 3457 to 2499 m^2^/m^2^. In addition, RPUF-3 formed the largest amount of char residue, which was dense and continuous enough to protect the polymer matrix from external heat and gas. Therefore, RPUF-3 could have excellent flame-retardant properties. In addition, the tensile strength, elongation at break, and compressive strength of RPUF-3 were also obviously improved.

## Figures and Tables

**Table 1:** LOI and UL-94 ratings of RPUF and its composites.

Sample	LOI/%	UL-94
RPUF-0	18.4	NR
RPUF-1	25.7	V-2
RPUF-2	25.5	V-2
RPUF-3	26.5	V-0

**Table 2:** The characteristic data tested via the cone calorimeter.

Samples	RPUF-0	RPUF-1	RPUF-2	RPUF-3
PHRR/kW·m^−2^	84.32	33.78	62.6	19.6
T_PHRR_/s	107	186	106	194
FGI/(kW/(m^2^·s))	0.79	0.18	0.60	0.10
THR/MJ·m^−2^	18.46	13.33	16.71	9.16
Y_CO2_/%	0.089	0.039	0.084	0.035
Y_CO_/%	0.004	0.009	0.006	0.008
TSP (m^2^/m^2^)	2589	3457	3276	2499
PSPR (m^2^/s)	64.89	50.37	56.79	50.53
Residues/%	4.0	22.2	18.2	24.6

**Table 3:** Thermogravimetric analysis data of RPUF and its composites.

Samples	T_-5 wt%_ (°C)	T_-10 wt%_ (°C)	T_-50 wt%_ (°C)	750 °C (wt%)
RPUF-0	198	259	403	3.3
RPUF-1	161	224	403	25.0
RPUF-2	168	242	345	23.7
RPUF-3	156	219	406	27.0

Note: T_-5 wt%_, T_-10 wt%_, and T_-50 wt%_ refer to a temperature of mass loss of 5 wt%, 10 wt%, and 50 wt%, respectively.

**Table 4:** Tensile strength, elongation at break, and compressive strength of RPUF and its composites.

Sample	Tensile Strength (Mpa)	Elongation at Break (%)	Compressive Strength (MPa)	Density (kg/m^3^)
RPUF-0	0.16 ± 0.03	38.52 ± 0.09	3.45 ± 0.02	65.4 ± 3.0
RPUF-1	0.08 ± 0.03	23.26 ± 0.11	1.19 ± 0.04	68.2 ± 4.2
RPUF-2	0.07 ± 0.02	23.47 ± 0.06	1.12 ± 0.01	75.5 ± 2.1
RPUF-3	0.09 ± 0.02	27.63 ± 0.05	1.66 ± 0.05	84.2 ± 3.5

**Table 5:** Composition of RPUF and that of its composites.

Samples	Polyether Polyol (wt%)	Diisocyanate (wt%)	EG (wt%)	APP (wt%)	GO (wt%)	PA-GO (wt%)
RPUF-0	50	50	0	0	0	0
RPUF-1	44.0	44.0	9.0	3.0	0	0
RPUF-2	44.0	44.0	9.0	3.0	0.3	0
RPUF-3	44.0	44.0	9.0	3.0	0	0.3

## Data Availability

Not applicable.

## References

[1] Sergei V.L., Edward D.W. (2002). Thermal decomposition, combustion and fire-retardancy of polyurethanes—A review of the recent literature. Polym. Int..

[2] Verdolotti L., Lavorgna M., Maio E.D., Iannace S. (2013). Hydration-induced reinforcement of rigid polyurethane-cement foams: The effect of the co-continuous morphology on the thermal-oxidative stability. Polym. Degrad. Stab..

[3] Estravís S., Tirado-Mediavilla J., Santiago-Calvo M., Ruiz-Herrero J.L., Villafañe F., Rodríguez-Pérez M.Á. (2016). Rigid polyurethane foams with infused nanoclays: Relationship between cellular structure and thermal conductivity. Eur. Polym. J..

[4] Modesti M., Lorenzetti A., Simioni F., Checchin M. (2001). Influence of different flame retardants on fire behaviour of modified PIR/PUR polymers. Polym. Degrad. Stab..

[5] Levchik S.V., Weil E.D. (2004). Thermal decomposition, combustion and flame-retardancy of epoxy resins—A review of the recent literature. Polym. Int..

[6] Tang Z., Maroto-Valer M.M., Andresen J.M., Miller J.W., Listemann M.L., McDaniel P.L., Morita D.K., Furlan W.R. (2002). Thermal degradation behavior of rigid polyurethane foams prepared with different fire retardant concentrations and blowing agents. Polymer.

[7] Chen M.J., Lin Y.C., Wang X.N., Zhong L., Li Q.L., Liu Z.G. (2015). Influence of cuprous oxide on enhancing the flame retardancy and smoke suppression of epoxy resins containing microencapsulated ammonium polyphosphate. Ind. Eng. Chem. Res..

[8] Gao M., Chen S., Wang H., Chai Z.H. (2018). Design, preparation, and application of a novel, microencapsulated, intumescent, flame-retardant-Based mimicking mussel. ACS Omega.

[9] Wen Y., Cheng Z., Li W., Li Z., Liao D., Hu X., Hull T.R. (2018). A novel oligomer containing DOPO and ferrocene groups:synthesis, characterization, and its application infire retardant epoxy resin. Polym. Degrad. Stab..

[10] Tang G., Zhang R., Wang X., Wang B.B., Song L., Hu Y., Gong X.L. (2013). Enhancement of flame retardant performance of bio-based polylactic acid composites with the incorporation of aluminum hypophosphite and expanded graphite. J. Macromol. Sci. A..

[11] Shi L., Li Z.M., Xie B.H., Wang J.H., Tian C.R., Yang M.B. (2006). Flame retardancy of different-sized expandable graphite particles for high-density rigid polyurethane foams. Polym. Int..

[12] Duan H.J., Kang H.Q., Zhang W.Q., Ji X., Li Z.M., Tang J.H. (2014). Core-shell structure design of pulverized expandable graphite particles and their application in flame-retardant rigid polyurethane foams. Polym. Int..

[13] Duquesne S., Bras M.L., Bourbigot S., Delobel R., Vezin H., Camino G., Eling B., Lindsay C., Roels T. (2003). Expandable graphite: A fire retardant additive for polyurethane coatings. Fire Mater..

[14] Cai Y., Wei Q., Huang F., Lin S., Chen F., Gao W. (2009). Thermal stability, latent heat and flame retardant properties of the thermal energy storage phase change materials based on paraffin/high density polyethylene composites. Renew. Energy.

[15] Zhang P., Song L., Lu H., Wang J., Hu Y. (2010). The influence of expanded graphite on thermal properties for paraffin/high density polyethylene/chlorinated paraffin/antimony trioxide as a flame retardant phase change material. Energy Convers. Manag..

[16] Higginbotham A.L., Lomeda J.R., Morgan A.B., Tour J.M. (2009). Graphite oxide flame-retardant polymer nanocomposites. ACS Appl. Mater. Interfaces.

[17] Yu B., Wang X., Qian X., Xing W., Yang H., Ma L., Lin Y., Jiang S., Song L., Hu Y. (2014). Functionalized graphene oxide/phosphoramide oligomer hybrids flame retardant prepared via in situ polymerization for improving the fire safety of polypropylene. RSC Adv..

[18] Wang X., Kalali E.N., Wan J.T., Wang D.Y. (2017). Carbon-family materials for flame retardant polymeric materials. Prog. Polym. Sci..

[19] Sang B., Li Z.W., Li X.H., Yu L.G., Zhang Z.J. (2016). Graphene-based flame retardants: A review. J. Mater. Sci..

[20] Jamsaz A., Goharshadi E.K. (2022). Graphene-based flame-retardant polyurethane: A critical review. Polym. Bull..

[21] Liao S.H., Liu P.L., Hsiao M.C., Teng C.C., Wang C.A., Ger M.D., Chiang C.L. (2012). One-step reduction and functionalization of graphene oxide with phosphorus-based compound to produce flame-retardant epoxy nanocomposite. Ind. Eng. Chem. Res..

[22] Long J., Liang B., Wang Z. (2020). Enhanced mechanical properties of APP flame-retardant epoxy resin by phosphorus-modified graphene oxide. Plast. Rubber Compos..

[23] Huang N.J., Cao C.F., Li Y., Zhao L., Zhang G.D., Gao J.F., Guan L.Z., Jiang J.X., Tang L.C. (2019). Silane grafted graphene oxide papers for improved flame resistance and fast fire alarm response. Compos. Part B Eng..

[24] Li K.Y., Kuan C.F., Kuan H.C., Chenc C.H., Shen M.Y., Yang J.M. (2014). Preparation and properties of novel epoxy/graphene oxide nanosheets (GON) composites functionalized with flame retardant containing phosphorus and silicon. Mater. Chem. Phys..

[25] Li Z.Q., Li W., Liao L., Li J.B., Wu T., Ran L.C., Zhao T.B., Chen B.S. (2020). Preparation and properties of polybutylene-terephthalate/graphene oxide in situ flame-retardant material. J. Appl. Polym. Sci..

[26] Gao Y.Y., Deng C., Du Y.Y., Huang S.C., Wang Y.Z. (2019). A novel bio-based flame retardant for polypropylene from phytic acid. Polym. Degrad. Stab..

[27] Zhang B., Yang S.J., Liu M.R., Wen P.Y., Liu X.Y., Tang G., Xu X.R. (2022). Bio-based trivalent phytate: A novel strategy for enhancing fire performance of rigid polyurethane foam composites. J. Renew. Mater..

[28] Otieno G., Kim J. (2008). Conductive graphite/polyurethane composite films using amphiphilic reactive dispersant: Synthesis and characterization. J. Ind. Eng. Chem..

[29] Zhang D.D., Zu S.Z., Han B.H. (2009). Inorganic-organic hybrid porous materials based on graphite oxide sheets. Carbon.

[30] Fang Y., Wang Q., Bai X., Wang W., Cooper P.A. (2012). Thermal and burning properties of wood flour-poly(vinyl chloride) composite. J. Therm. Anal. Calorim..

[31] Gu J., Zhang G., Dong S., Zhang Q., Kong J. (2007). Study on preparation and fire-retardant mechanism analysis of intumescent flame-retardant coatings. Surf. Coat. Technol..

[32] Wei W.C., Deng C., Huang S.C., Wei Y.X., Wang Y.Z. (2018). Nickel-Schiff base decorated graphene for simultaneously enhancing the electroconductivity, fire resistance, and mechanical properties of polyurethane elastomer. J. Mater. Chem. A.

[33] Yuan Y., Wang W., Shi Y.Q., Song L., Ma C., Hu Y. (2020). The influence of highly dispersed Cu_2_O-anchored MoS_2_ hybrids on reducing smoke toxicity and fire hazards for rigid polyurethane foam. J. Hazard. Mater..

[34] Gao M., Li J.F., Zhou X. (2019). A flame retardant rigid polyurethane foam system including functionalized graphene oxide. Polym. Compos..

[35] Hummers W.S., Offeman R.E. (1958). Preparation of Graphitic Oxide. J. Am. Chem. Soc..

